# Systematic unravelling of the biosynthesis of poly (L-diaminopropionic acid) in *Streptomyces albulus* PD-1

**DOI:** 10.1038/srep17400

**Published:** 2015-12-03

**Authors:** Zhaoxian Xu, Zhuzhen Sun, Sha Li, Zheng Xu, Changhong Cao, Zongqi Xu, Xiaohai Feng, Hong Xu

**Affiliations:** 1 State Key Laboratory of Materials-Oriented Chemical Engineering, Nanjing, 211816, China; 2 College of Food Science and Light Industry, Nanjing Tech University, Nanjing, 211816, China

## Abstract

Poly(L-diaminopropionic acid) (PDAP) is one of the four homopoly(amino acid)s that have been discovered in nature. However, the molecular mechanism of PDAP biosynthesis has yet to be described. In this work, the general layout of the PDAP biosynthetic pathway is characterised in *Streptomyces albulus* PD-1 by genome mining, gene disruption, heterologous expression and *in vitro* feeding experiments. As a result, L-diaminopropionic acid (L-DAP), which is the monomer of PDAP, is shown to be jointly synthesised by two protein homologues of cysteine synthetase and ornithine cyclodeaminase. Then, L-DAP is assembled into PDAP by a novel nonribosomal peptide synthetase (NRPS) with classical adenylation and peptidyl carrier protein domains. However, instead of the traditional condensation or thioesterase domain of NRPSs, this NRPS has seven transmembrane domains surrounding three tandem soluble domains at the C-terminus. As far as we know, this novel single-module NRPS structure has only been reported in poly(ε-L-lysine) synthetase. The similar NRPS structure of PDAP synthetase and poly(ε-L-lysine) synthetase may be a common characteristic of homopoly(amino acid)s synthetases. In this case, we may discover and/or design more homopoly(amino acid)s by mining this kind of novel NRPS structure in the future.

In recent years, the homopoly(amino acid)s secreted by microorganisms have been extensively investigated because of their unique characteristics. Homopoly(amino acid)s are distinguished from common peptides and proteins because homopoly(amino acid)s are composed of a single type of amino acids^1^. To date, more than 300 types of amino acids have been discovered. However, only four homopoly(amino acid)s, namely, poly(γ-glutamic acid) (γ-PGA), poly(ε-L-lysine) (ε-PL), poly(γ-L-diaminobutanoic acid) (γ-PAB) and poly(L-diaminopropionic acid) (PDAP) have been found in nature^1^,^2^,^3^ (Fig. 1). The scarcity of homopoly(amino acid)s is probably due to the lack of suitable synthetase that can assemble amino acid monomers into homopoly(amino acid)s. Among the four kinds of homopoly(amino acid)s, γ-PGA and ε-PL have many applications; for example, γ-PGA has been broadly used in foods, cosmetics, medicines, water treatment and agriculture^4^,^5^. ε-PL has also been extensively used in foods, medicines, new materials and electronics; furthermore, ε-PL has been approved as a natural food preservative in Japan, the United States, China, and other countries^6^,^7^.

In a previous study, PDAP, the fourth homopoly(amino acid)s, was purified and identified from the culture broth of *Streptomyces albulus* PD-1, a well-known ε-PL-producing strain. Structurally, PDAP is an L-α,β-diaminopropionic acid (L-DAP) oligomer that links the amine functional group and the carboxylic acid functional group; this substance has a molecular mass distribution ranging from 0.5 to 1.5 kDa^3^. With a novel polycationic structure, PDAP, similar to ε-PL, can be regarded as a potentially specific and advanced platform for many fields. For example, it can be used as a natural food preservative, as a non-viral gene delivery vector or as a raw material for hydrogels. Despite their similarity, PDAP exhibits a higher charge density than ε-PL. Thus, PDAP is more suitable for some applications than ε-PL. As far as we know, PDAP shows efficient antibacterial activities against various microorganisms and exhibits stronger inhibitory activities against yeasts than ε-PL^3^. In our previous work, PDAP fermentation was optimised using a pH- and dissolved oxygen-controlled strategy; this optimised process yielded 9.6 g/L PDAP^8^. However, the genes or enzymes involved in PDAP biosynthesis remain unknown.

In previous studies, the enzymes involved in the biosynthesis of γ-PGA and ε-PL have already been described to some extent. In γ-PGA biosynthesis, two different assembly modes, the thiotemplate mechanism catalysed by non-ribosomal peptide synthetases (NRPSs) and the amide-ligation mechanism catalysed by amide ligases, have been postulated. In the thiotemplate mechanism, L-glutamic acid is initially activated by ATP; this substance then binds to a sulfhydryl group and isomerises into a D-form, and the isomerised product is subsequently elongated to form the D-homopolymer of γ-PGA^9^. In the amide-ligation mechanism, ATP is transferred to activate the terminal carboxyl group of a growing γ-PGA chain. Subsequently, a new amide linkage is formed via nucleophilic attack by the amino group of glutamic acid to elongate the γ-PGA chain to the D/L-homopolymer^5^,^10^. In ε-PL, L-lysine is catalysed to ε-PL by an unusual NRPS comprised of an adenylation domain (A domain), a peptidyl carrier protein domain (PCP domain) and three special tandem domains surrounding six transmembrane (TM) domains^11^. In this process, an L-lysine monomer is initiated in the N-terminus by the adenylated A domain and the resulting lysyl-O-AMP is transferred to the PCP domain. Then, the unusual C-terminal tandem domains catalyse peptide bond formation and iteratively extend the biopolymer. Such work raises the following interesting questions: What is the structure of the PDAP synthetase (PDAPs)? How does it assemble L-DAP into a biopolymer? Is there any similarity between the PDAPs and the reported homopoly(amino acid)s synthetases?.

In this study, candidate genes for the *de novo* biosynthesis of PDAP were predicated from *S. albulus* PD-1 by sequencing and annotating its genome. The putative genes were identified and characterised using genetic inactivation, heterologous expression and biochemical assays. In particular, the PDAPs was found to be a novel NRPS with a typical A domain, a typical PCP domain and seven TM domains surrounding three tandem soluble domains in the C-terminus. To our knowledge, this novel single-module NRPS structure has only been reported in the poly(ε-L-lysine) synthetase (Pls). Our findings form the basis for genetic and biochemical studies into PDAP biosynthesis. Moreover, the similar NRPS structure of PDAPs and Pls provides us insight into the discovery and/or design of numerous homopoly (amino acid)s in the future.

## Results and Discussion

### Prediction and identification of the PDAP synthetase

**Figure 2:**
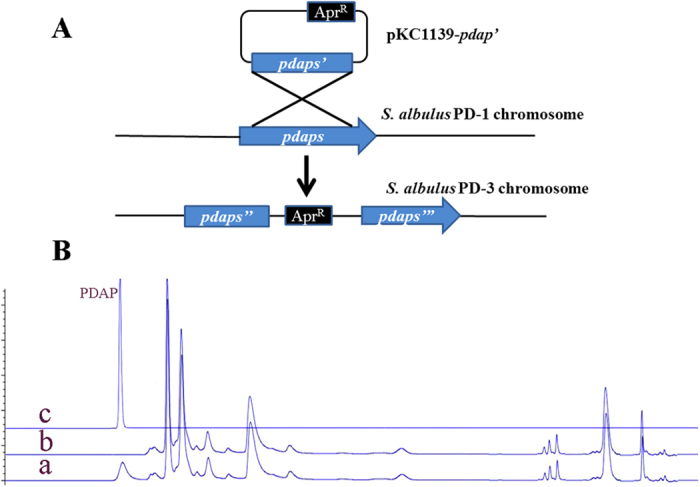
Construct used to obtain the loss-of-function mutants. (**A**) Diagram of putative *pdaps* disruption. (**B**) HPLC analysis of the culture of the predicted PDAP-inactivated strain: a, culture broth of the wild-type *S. albulus* PD-1 strain; b, culture broth of the predicted PDAPs-inactivated strain; c, a PDAP standard.

Peptide natural products are commonly synthesised via either ribosomal or non-ribosomal mechanisms^1^,^12^. In contrast with the ribosomal machinery and its 21 amino acids building blocks for protein biosynthesis, the non-ribosomal machinery can accept many non-proteinogenic amino acid building blocks as substrates. PDAP is a biopolymer comprised of L-DAP, a novel non-proteinogenic amino acid. Thus this biopolymer is most likely assembled by an NRPS. Recently, with the rapid development of genome sequencing technologies and bioinformatics, an efficient platform for the discovery and identification of new metabolic pathways has been established via genome mining^13^,^14^,^15^,^16^,^17^. For example, the genetic variations associated with ε-PL biosynthesis in *S. albulus* ZPM have been identified through genome sequencing^18^. A novel xylose isomerase in the xylose utilisation pathway in *Bacillus coagulans* has also been identified through genome analysis^17^. Therefore, we initially determined the biosynthetic pathway of PDAP based on the genome data of *S. albulus* PD-1^19^. We investigated the *S. albulus* PD-1 genome and found eight NRPS genes. The substrate specificities of these NRPSs were predicted using NRPSpredictor2. The *in silico* analysis results are listed in Table 1. β-lysine, the substrate of gb|EXU85975.1|, which is an aliphatic amino acid with an amino group at the β position, is most similar to L-DAP in structure (Supplementary Fig. S1). Thus, gb|EXU85975.1| was considered the most likely PDAPs. To verify this hypothesis, we constructed a gene disruption mutant of the putative *pdaps* gene(Fig. 2A). The *pdaps* disruption was confirmed by PCR and Southern blot hybridization (Supplementary Fig. S2). Then the culture broth of the disruptant was detected. As shown in Fig. 2B, the disruption of the putative *pdaps* terminated PDAP production, and this finding provided strong evidence that PDAPs had been correctly identified. Moreover, the mutant strains exhibited poorer spore production activity than wild-type strains.

**Figure 3:**
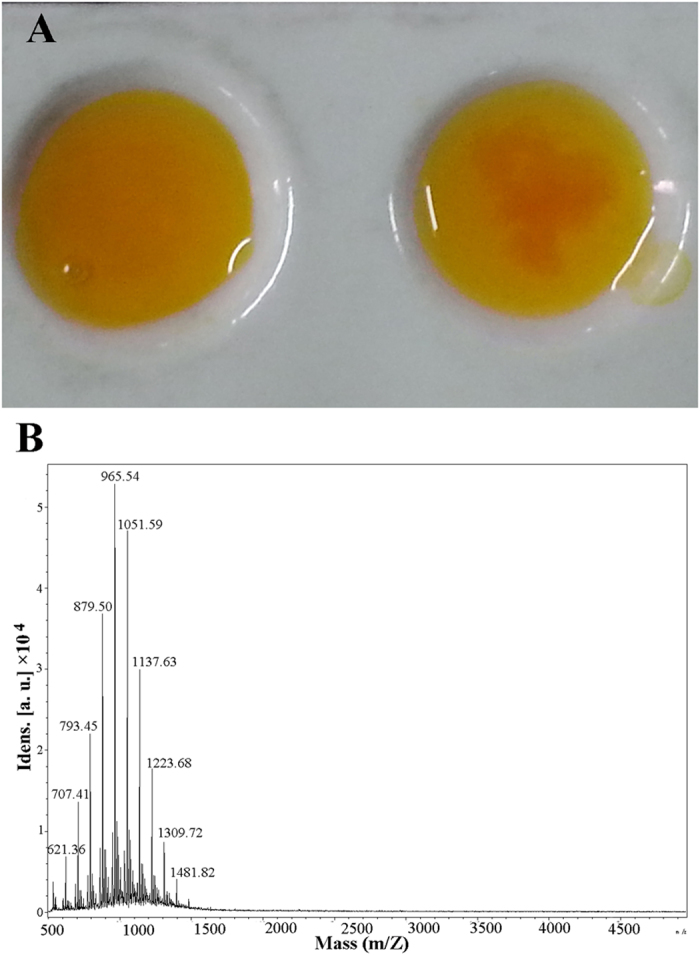
(**A**) Reaction between culture supernatant and Dragendorff reagent: Culture supernatant of (**a**) *S. lividans* TK24 and (**b**) *S. lividans* TK24-*pdaps*. (**B**) PDAP was purified from the culture broth of *S. lividans* TK24-*pdaps* and subsequently analysed through MALDI-TOF/MS. The regular peak at an equal interval is 86.05, which is equal to the molecular of L-DAP (104.07) deducts the molecular of H2O (18.02).

To further verify the identity of the hypothesised PDAPs, the target gene was expressed in a heterologous host, and an *in vitro* feeding experiment was conducted. In previous studies, the ε-PL synthetase gene (*pls*) was expressed via the control of a constitutive promoter; however, neither Pls expression nor ε-PL production was observed. Ultimately, the Pls was successfully expressed in *S. albulus* and *S. lividans* under the control of its own promoter^20^,^21^. The researchers posited that the Pls expression was probably strictly controlled because Pls is a membrane protein and the constitutive promoter was not suitable for the transcription of Pls. Because similar TM domains also existed in PDAPs, we expressed PDAPs via the control of its own promoter in *S. lividans* TK24, a model streptomycete commonly used as a host strain for heterologous expression. Positive colonies were selected and cultured in M3G medium supplemented with L-DAP (1 g/L) for one week; subsequently, the supernatant of the culture broth was characterised via high-performance liquid chromatography (HPLC). However, not any significant characteristic peak was detected through HPLC possibly because of the low concentration (<0.05 g/L) of PDAP produced by the recombinant strain. In contrast, the culture broth supernatant tested positive with Dragendorff reagent, which is frequently used to screen alkaloids (Fig. 3A). Thus, the culture broth supernatant from the recombinant strain was concentrated, purified and evaluated with matrix-assisted laser desorption ionisation-time of flight mass spectrometry (MALDI-TOF/MS) using a spectrometer (Autoflex 2, Bruker Daltonics Inc.,USA). The results revealed the presence of a polydisperse PDAP group (Fig. 3B); this group exhibited the same molecular weight as previously reported^3^. Gene interruption, heterologous expression and *in vitro* feeding tests rigorously demonstrated that the identified novel NRPS (gb|EXU85975.1|) was indeed the PDAPs.

### Bioinformatic analysis of the PDAP synthetase

Based on the genome sequence of *S. albulus* PD-1, the PDAPs is encoded by a single ORF of 4,338 bp. With the nucleotide sequence and protein sequence in hand, the structure and function of the enzyme were investigated. In general, NRPSs contain three core domains. The A domain selects and activates the candidate amino acid as an amino acyladenylate. Then, the PCP domain covalently binds the activated monomer via a phosphopantetheinyl arm. The C domain then catalyses the formation of the peptide linkage between the activated amino acids from two adjacent PCP modules. These three domains constitute the repeating module responsible for the activation and incorporation of an amino acid into the growing peptide chain. Finally, a mature peptide is released from the PCP domain, and this release is catalysed by a C-terminal thioesterase (TE) domain^22^,^23^,^24^. The conserved domains of PDAPs were analysed, and the results revealed that PDAPs exhibited an unusual NRPS structure, with a classical A domain and a classical PCP domain in the N-terminus. However, PDAPs did not contain a domain exhibiting significant sequence similarity to the traditional C domains that are essential for peptide bond formation in NRPSs. Furthermore, PDAPs did not contain a traditional TE domain, which catalyses the release of the final product from NRPS enzymes. Instead, a NRPS terminal domain with an unknown function was found in the C-terminal region of PDAPs. This identified domain was approximately 779 amino acids in length and roughly thrice the size of a traditional TE domain. Physicochemical analysis suggested that seven TM domains were present in this novel domain and this novel domain was divided into three subdomains on the basis of the location of the TM domains (Supplementary Fig. S3). A sequence alignment revealed that the three subdomains displayed substantial sequence similarity (with pairwise identities of 23.1%, 26.1% and 22.6%; Supplementary Fig. S4A). In fact, the structure with three tandem soluble domains surrounded by several TM domains has only been reported in Pls; all three tandem domains were essential for peptide bond formation in ε-PL biosynthesis^11^,^25^. Given that these sequences may play the same role in peptide bond formation in PDAPs, we named theses tandem sequences the C1, C2 and C3 domain, similar to those found in Pls. In fact, multiple sequence alignments of the C1, C2, and C3 domains in PDAPs and Pls revealed a few universally conserved amino acids in their structures (Supplementary Fig. S4B). The similar NRPS structures of PDAPs and Pls may be a common characteristic of homopoly(amino acid)s synthetase. For further, this novel type NRPS was mined from the genome database, and many NRPSs with similar structure were discovered, for example, ref|WP_052230086.1|, gb|AJE44528.1|, gb|AHH98491.1|. All of these proteins are presumed to be related to peptide synthesis, but none of them was studied intensively. This is a great resource for homopoly(amino acid)s investigation. As such, we can discover more homopoly(amino acid)s by mining for this novel type of NRPS in the future.

**Figure 4:**
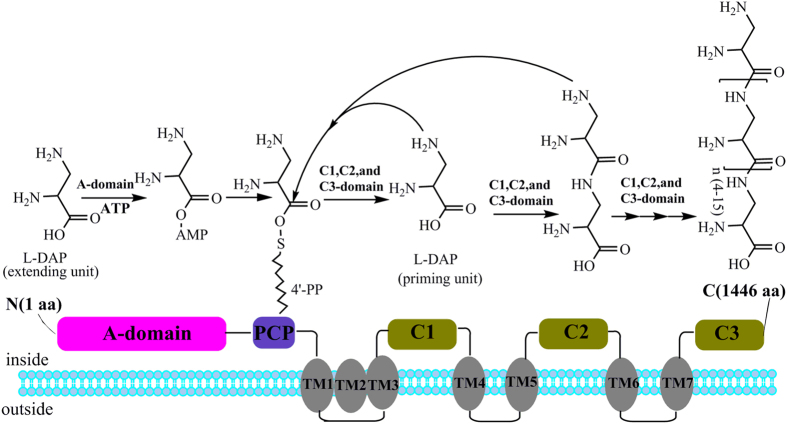
Domain architecture and predicted mechanism of PDAPs. A domain, PCP domain, seven TM domains and three tandem domains (C1, C2 and C3 domains) are shown schematically.

On the basis of the structure of PDAPs, we predicted the mechanism of PDAPs. As shown in Fig. 4, L-DAP is initially activated by an A domain, the active L-DAP monomer is transferred by PCP domain, and three unusual C-terminal tandem domains catalyse peptide bond formation and iteratively extend the polymer. However, several factors remain unknown. For example, a traditional TE domain was not detected in PDAPs; further studies have yet to determine the mechanism by which the release of peptides are catalysed and the conditions that yield a PDAP molecular mass distribution from 0.5 to 1.5 kDa.

### Substrate specificity analysis of the adenylation domain of PDAPs

**Figure 5:**
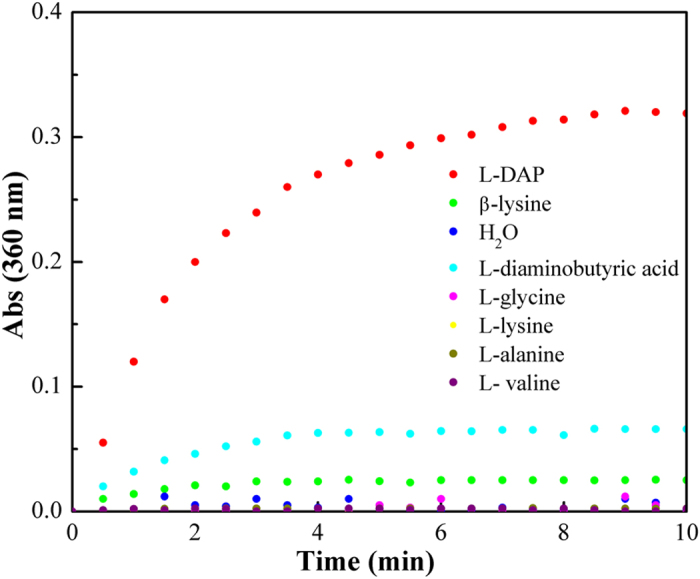
Substrate specificity of the A domain of PDAPs. Substrate specificity was indicated by the released PPi level.

Given that PDAPs can accept L-DAP as a substrate, we determined whether this novel NRPS could tolerate additional substrate diversity. In NRPS modules, the A domains select and activate the incoming monomeric amino acids^26^. Hence, the substrate specificity of the A domain of PDAPs was investigated. The amino acid-dependent PPi release assay was conducted to evaluate the substrate specificity of the A domain. As a result, the highest PPi level was detected using L-DAP as a substrate (Fig. 5); no other adenylation activity was observed when L-DAP was replaced with L-lysine, L-alanine, L-valine or L-glycine. Moreover, the adenylation activity was weaker when L-diaminobutyric acid or β-lysine was used as a substrate than when L-DAP was used. However, no significant product was detected via HPLC or Dragendorff reagent when L-diaminobutyric acid or β-lysine was fed to *S. lividans* TK24-*pdaps*. Thus, other portions of PDAPs may exert regulatory selection outside of the A domain. In general, the results of the PPi release assay were slightly different from those of the *in silico* prediction from NRPSpredictor2, which suggested that the preferred substrate of the A domain is β-lysine. As noted before, the NRPS predictor yields suboptimal results when small substrates are used^27^; thus it is reasonable that the predicted results are slightly inconsistent with our biochemical assay results because L-DAP is a small amino acid.

### Identification of the L-DAP biosynthetic pathway

In the above work, PDAP was shown to be biosynthesised from L-DAP through a novel NRPS in *S. albulus* PD-1. Then, the biosynthetic pathway of L-DAP was identified in the following work. In previous studies, L-DAP has been reported to be a precursor of some antibiotics, such as viomycin, the capreomycins, and zwittermicin A^28^,^29^,^30^,^31^. In addition, L-DAP is also a precursor molecule of staphyloferrin B, one of the two siderophores in *Staphylococcus aureus*^32^,^33^. In the past decades, the biosynthetic pathway of L-DAP in some strains has been investigated step by step. Precursor labelling has been applied to the capreomycins and viomycin biosynthetic pathways, and results have demonstrated that L-serine is the precursor of L-DAP^28^,^29^. Subsequently, experiments and the analysis of the gene clusters of viomycin, the capreomycins, zwittermicin A and staphyloferrin B suggest that L-DAP biosynthesis is catalysed by the concerted actions of two enzymes^30^,^31^,^32^,^33^,^34^. Based on the bioinformatic analysis of these two enzymes, several proposed mechanisms were proposed for L-DAP biosynthesis. Recently, Marek *et al.* first confirmed O-phospho-L-serine and L-glutamate are the substrates of L-DAP formation in *Staphylococcus aureus*^35^. On the basis of these findings, we assumed that L-DAP biosynthesis in *S. albulus* PD-1 may follow the same pathway and the proteins involved in L-DAP synthesis were mined from the genome data of *S. albulus* PD-1.

**Figure 6:**
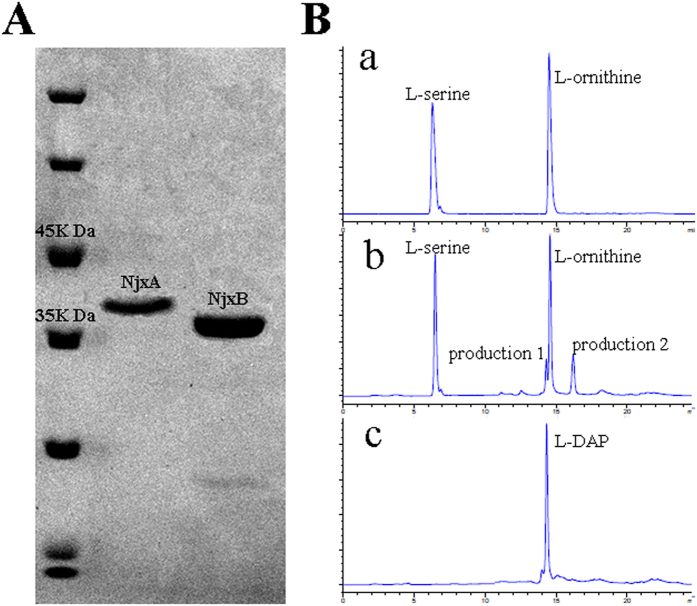
(I) SDS-PAGE analysis of purified NjxA and NjxB. M, protein molecular weight marker; 1, purified NjxA 2, purified NjxB. (II) HPLC for the recombinant enzymatic activities of NjxA and NjxB: a, the reaction mixture without NjxA and NjxB; b, reaction mixture with NjxA and NjxB; c, a L-DAP standard.

**Figure 7:**
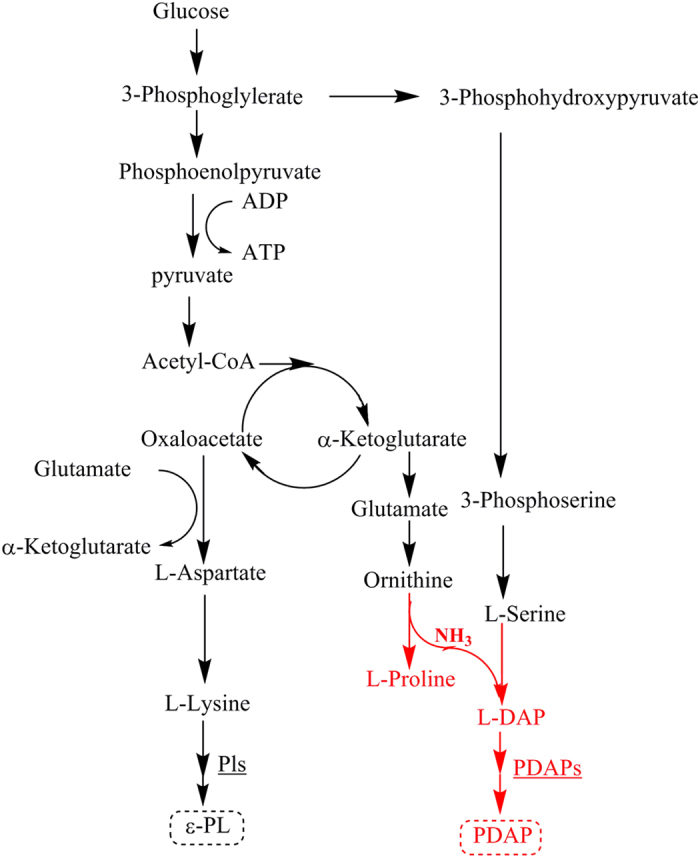
The basic metabolic pathway of *S. albulus* PD-1. The red part indicates the pathway involved in PDAP biosynthesis.

Two proteins encoded by adjacent genes were regarded as the most probable L-DAP synthetase candidantes on the basis of sequence similarity with the reported L-DAP synthetase, namely, NjxA (gb|EXU92816.1|) and NjxB (gb|EXU92121.1|) (Supplementary Table S2). To further confirm the function of these two enzymes, NjxA and NjxB were expressed and purified and then used to jointly perform a catalytic reaction *in vitro* (Fig. 6). However, when O-phospho-L-serine and L-glutamate were used as substrates, no L-DAP production was detected. These results indicated that L-DAP biosynthesis in *S. albulus* PD-1 may be somewhat different from that in *Staphylococcus aureus*. Thus, other substrates proposed in previous studies were added to the reaction mixture^30^,^33^. Fortunately, a significant peak of L-DAP was observed when L-serine and L-ornithine were used as substrates (Fig. 6B). These results were little different than those reported by Marek *et al.* and the L-DAP in *S. albulus* PD-1 appears to be biosynthesised via the mechanism proposed by Michael *et al.* In this manner, NjxA acts as the cysteine synthase and serine dehydrate, which first catalyses L-serine to form a pyridoxal phosphate (PLP)-bound α-aminoccrylate intermediate; then it catalyses a β-substitution replacement reaction to generate L-DAP from the α-aminoccrylate intermediate using ammonia as a nucleophile, which is released when NjxB catalyses the cyclisation of L-ornithine (Supplementary Fig. S5). The difference between L-DAP biosynthesis in *S. aureus* and *S. albulus* PD-1 may be due to the adaptive evolution of these two different species. In *S. aureus*, SbnA and SbnB catalyse O-phospho-L-serine and L-glutamate to α-oxoglutarate and L-DAP, which not only provide two of the three precursors required for staphyloferrin B biosynthesis in *S. aureus*, but also lessen dependence on tricarboxylic acid (TCA) cycle metabolites for staphyloferrin B biosynthesis during iron deprivation. For *S. albulus* PD-1, a powerful TCA cycle was detected, and thus more metabolic precursors may be available for L-DAP and PDAP biosynthesis from the TCA cycle^36^,^37^. Thus, we can conclude that *S. albulus* PD-1 synthesises PDAP in the following manner: L-DAP is initially synthesised from L-serine and L-ornithine and subsequently assembled into PDAP by a novel NRPS. Although the genes responsible for many secondary metabolites are usually clustered together in the genome^38^,^39^, the genes implicated in this L-DAP biosynthesis pathway are not located close to *pdaps*. In this study, the biosynthetic pathway of PDAP in *S. albulus* PD-1 was elucidated in detail and it is an important supplement to the basic metabolic network in *S. albulus* PD-1(Fig. 7). Our findings provide a solid basis for the production of PDAP and ε-PL by *S. albulus* PD-1. In particular, the novel structure of PDAPs is a relevant reference for the discovery and design of numerous homopoly(amino acid)s in the future.

## Methods

### Strains, plasmid and media

*S. albulus* PD-1 (deposited in the China Centre for Type Culture Collection with the strain number M2011043), a well-known ε-PL- and PDAP-producing strain, was used in this study. *E. coli* ET12567 (pUZ8002) was used as a donor during conjugal transfer. pKC1139 was used to disrupt the putative *pdaps* gene. pIJ86 was used for heterologous expression of the putative PDAPs. The pET22b plasmid was used to facilitate the expression of the two putative key enzymes involved in L-DAP biosynthesis. The details of the strains, plasmids and media used in this study are listed in Supplementary Table S1.

### Gene prediction and bioinformatic analysis

The genome sequence of *S. albulus* PD-1 was annotated using the NCBI Prokaryotic Genomes Automatic Annotation Pipeline (http://www.ncbi.nlm.nih.gov/genome/annotation_prok/)^19^. The conserved domains of the candidate proteins were identified using the Conserved Domain Database of NCBI (http://www.ncbi.nlm.nih.gov/cdd/)^40^. The sequence alignment was performed using the DNAMAN program and the BLAST module in NCBI. The substrate specificities of the NRPS A domains were predicted by using NRPSpredictor2 (http://nrps.informatik.uni-tuebingen.de/Controller? cmd=SubmitJob)^41^. The TM domains were determined using TMHMM Server v.2.0 (http://www.cbs.dtu.dk/services/TMHMM/)^42^.

### Inactivation and heterologous expression of the putative *pdaps* gene

The target *pdaps* gene was inactivated through single crossover homologous integration with an apramycin resistance cassette as a selective marker (Fig. 2A). A 3.2-kb homologous fragment of *pdaps* was amplified from *S. albulus* PD-1, digested with *EcoR*I and *Hin*dIII and ligated into pre-digested pKC1139 harbouring the apramycin resistance cassette. The resulting plasmid was used for transformation via an optimised conjugal transfer method. The exconjugant was grown at 30 °C on MS medium containing apramycin (50 μg/mL) and nalidixic acid (20 μg/mL). The exconjugant strain was then inoculated into liquid tryptone soy broth (TSB) medium and cultivated in a shake flask at 30 °C and 200 rpm. After 24 h, the culture broth was streaked on an MS agar plate containing 50 μg/mL apramycin and cultivated at 37 °C to obtain the putative gene disruptants.

To confirm whether pKC1139-*pdaps* was successfully inserted into the chromosomal copy of *pdaps*, we extracted and analysed the chromosomal DNA from the mutant strains through PCR amplification using start and the end sequences of putative *pdaps* as primers. In addition, Southern blot was also applied to confirm the disruption of the putative *pdaps* by using apramycin-resistant gene as a probe. To confirm whether the mutant strains could produce PDAP, we cultivated spores of the recombinants in M3G medium and in a shake flask at 30 °C and 200 rpm for 72 h. The culture filtrates were characterised using HPLC with an Agilent 1200 instrument and according to a previously described method^3^.

To achieve the heterologous expression of PDAPs in *S. lividans* TK24, the intact *pdaps* gene with the corresponding native promoter was amplified with specific primers (Supplementary Table S2). The PCR products were digested with *Bam*HI and *Hin*dIII and subsequently inserted into pIJ86 to generate pIJ86-*pdaps*. pIJ86-*pdaps* was transformed into *E. coli* ET12567 (pUZ8002) and then transferred into *S. lividans* TK24 by the standard method described in *Practical Streptomyces Genetics*^43^. The transformants were selected and detected through colony PCR. The spores of the candidate transformants were cultured at 30 °C for 72 h in M3G medium supplemented with 1 g/L L-DAP. The supernatant of the culture broth was tested with Dragendorff reagent and characterised with HPLC for PDAP production.

### Analysis of the *in vitro* substrate specificity of the PDAPs adenylation domain

The A domain-encoding fragment was amplified from *S. albulus* PD-1 with the primers listed in Supplementary Table S3. The PCR fragment was digested with *EcoR*I and *Hin*dIII and inserted into the corresponding sites of pET28a; as a result, pET28a-*pdapsa* was generated. The recombinant plasmid was introduced into *E. coli* BL21 (DE3) to overexpress the corresponding protein. The expressed protein was purified through Ni-chelating affinity chromatography.

In the first step of an NRPS assembly line, a specific amino acid is selected by the A domain. A domains consume ATP to activate amino acid substrates, with concomitant pyrophosphoric acid (PPi) release^24^. Thus, the released PPi can be used as an indicator of the substrate specificity of an A domain. For PDAPs, the PPi levels were measured through a continuous spectrophotometric assay with the EnzChek pyrophosphate assay kit (Molecular Probes)^31^. The reactions were conducted at 30 °C in a 500 μL reaction mixture that contained 1 × buffer, 0.2 mM MesG, 10 mM MgCl_2_, 5 mM ATP, 10 mM amino acid, 0.3 U inorganic pyrophosphatase, 1 U purine nucleoside phosphorylase and 50 μL A domain. The reaction mixture was monitored using a microplate reader (Thermo, Fisher), and readings were conducted every 30 s for 10 min at a wavelength of 360 nm.

### Identification of the L-DAP biosynthesis pathway

Two candidate genes responsible for the biosynthesis of L-DAP were identified based on a genetic similarity analysis: *njx A* and *njx B*. Both genes were amplified via PCR from the genomic DNA of *S. albulus* PD-1. The upstream and downstream oligonucleotide primers for *njxA* and *njxB* are listed in Supplementary Table S3. Then, the PCR fragments were cloned into the *EcoR*I/*Hin*dIII restriction sites of the pET-22b plasmid. The recombinant plasmid pET-22b-*njxA* and pET-22b*-njxB* were verified through restriction enzyme digestions and DNA sequencing (GenScript Biotech Co., China). The recombinant plasmids were transformed into *E. coli* BL21 (DE3), *E. coli* Rosetta (DE3) and *E. coli* Origami (DE3); *E. coli* Origami (DE3) was selected as the best host for the co-expression of *njx A* and *njx B* because more soluble NjxA and NjxB protein could be obtained when expressed in this strain.

Expression studies were conducted in *E. coli* Origami (DE3). A single colony from freshly transformed cells was inoculated into 5 mL LB medium supplemented with 50 μg/mL ampicillin and grown at 37 °C for 8 to 10 h. Then, 1 mL of the cell suspension was incubated in 50 mL LB supplemented with 50 μg/mL ampicillin and was subsequently incubated at 37 °C. When the OD_600_ reached 0.6, isopropyl-β-D-thiogalactopyranoside was added to a final concentration of 1 mM, and the culture was incubated for 20 h at 15 °C to induce protein expression. The induced cells were harvested through centrifugation and suspended in normal saline. The cells were then disrupted through sonication in an ice bath, and the expressed proteins were purified through Ni-chelating affinity chromatography. The recombinant enzymatic activities were determined by measuring the amount of L-DAP in a reaction mixture. The reaction mixture contained L-serine (1 g/L), L-ornithine (1 g/L), PLP (50 mg/L), NAD^+^ (50 mg/L), purified NjxA (50 μL) and purified NjxB (50 μL) in a total volume of 200 μL (Tris-HCl, pH 7.5). The reaction mixture was incubated at 37 °C for 30 min and evaluated through HPLC. Online derivatisation was performed using orthophthalaldehyde for the primary amino acids and 9-fluorenylmethyl chloroformate for the secondary amino acids. The amino acid derivatives were analysed using an Agilent 1200 series HPLC system.

## Additional Information

**How to cite this article**: Xu, Z. *et al.* Systematic unravelling of the biosynthesis of poly (L-diaminopropionic acid) in *Streptomyces albulus* PD-1. *Sci. Rep.* **5**, 17400; doi: 10.1038/srep17400 (2015).

## Supplementary Material

Supplementary Information

## Figures and Tables

**Figure 1:**
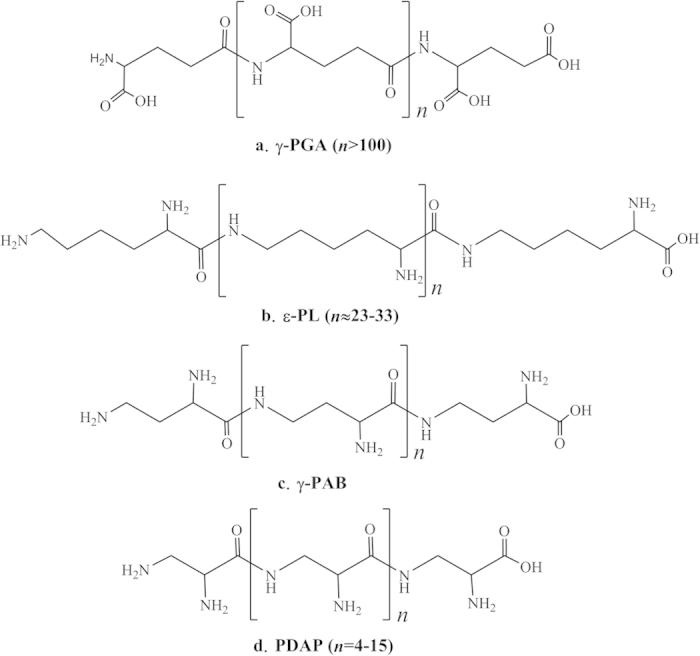
Chemical structures of the discovered homopoly(amino acid)s. (**a**) γ-PGA, (**b**) ε-PL, (c) γ-PAB, and (d) PDAP.

**Table 1:** The substrate specificity of those NRPSs in *S. albulus* PD-1.

Sequence ID	Substrate cluster description	Nearest neighbor target specificities
gb|EXU92123.1|	Aliphatic side chain with B-bond donor	glutamine
gb|EXU92158.1|	Aliphatic side chain with B-bond donor	lysine
gb|EXU92697.1|	Aliphatic side chain with B-bond donor	serine
	Apolar, aliphatic side chains	glycine
gb|EXU88741.1|	Aliphatic side chain with B-bond donor	asparagine
gb|EXU86680.1|	Apolar, aliphatic side chains	phenylalanine
gb|EXU85975.1|	Aliphatic, branched hydrophobic side chain	β-lysine
gb|EXU91762.1|	Aliphatic side chain with B-bond donor	glutamine
gb|EXU90606.1|	hydrophobic-aliphatic	lysine
