# Supplementary material for: Systematic unravelling of the biosynthesis of poly (L-diaminopropionic acid) in Streptomyces albulus PD-1
Source: Sci Rep. 2015 Dec 3;5:17400. doi: 10.1038/srep17400 (PMC4668381; doi:10.1038/srep17400)
Supplement: Supplementary Information [file srep17400-s1.pdf]

Supplementary Materials

Systematic unravelling of the biosynthesis of poly(L-diaminopropionic acid) in *Streptomyces albulus* PD-1

Zhaoxian Xu[1,2], Zhuzhen Sun[1,2], Sha Li[1,2], Zheng Xu[1,2], Changhong Cao[1,2], Zongqi Xu[1,2], Xiaohai Feng[1,2,*], Hong Xu[1,2,*]

[1]State Key Laboratory of Materials-Oriented Chemical Engineering, Nanjing, 211816, China

[2]College of Food Science and Light Industry, Nanjing Tech University, Nanjing, 211816, China

[*]Corresponding Authors: Prof. Hong Xu and Prof. Xiaohai Feng, (Tel/Fax: +86-25-58139433; E-mail address: xuh@njtech.edu.cn, fengxiaohai@njtech.edu.cn)

Supplementary Table S1. Strains and plasmids used in this study

Strains or plasmids	Descriptions	Source
S. albulus PD-1	Producer of ε-PL and PDAP	CGMCC No. M2011043
S. albulus PD-3	△*pdaps* :: Am^R	This study
S. lividans TK 24	A model streptomycete used for PDAPs expression	Lab stock
E. coli DH 5α	*E. coli* strain used for cloning	Lab stock
E. coli BL 21 (DE3)	*E. coli* strain used for the expression A domain of PDAPs	Lab stock
E. coli Origami (DE3)	*E. coli* strain used for expression of enzymes involved in L-DAP synthesis	Lab stock
pET-28a	Plasmid used for the expression A domain of PDAPs	Lab stock
pET-22b	Plasmid used for the expression of NjxA and NjxB	Lab stock
pKC1139	Used for the gene disruption of putative *pdaps*	Lab stock
pKC1139-*pdap'*	pKC1139 carrying homologous sequence of *pdaps*	This study
pIJ86	Used for the heterologous expression of PDAPs in *S. lividans* TK24	Lab stock

Supplementary Table S2. List of similarity between NjxA/NjxB and the reported proteins related to L-DAP biosynthesis

Organism	Homologous gene (NjxA/NjxB)	Similarity (%NjxA /%NjxB)	Query cover (NjxA%/NjxB%)	Related products	References
Streptomyces sp. strain ATCC 11861	VioB/VioK	33/31	84/87	viomycin	Antimicrobial agents and chemotherapy, 2003, 47(9): 2823-2830
S. mutabilis subsp. *capreolus* ATCC 23892	CmnB/CmnK	34/32	82/98	capreomycin	Applied and environmental microbiology, 2007, 73: 4162-4170
Bacillus thuringiensis sub. *kurstaki* YBT-1520	Zwa5A/Zwa5B	30/30	82/79	zwittermicin A	FEBS letters, 2008, 582: 3125-3131
Staphylococcus aureus Newman	SbnA/SbnB	31/27	95/90	staphyloferrin B	Molecular microbiology, 2009 74: 594-608

Supplementary Table S3. Sequences of primer pairs used in this study

Target products	Primer sequences (5′-3′)
Homologous fragment of *pdaps*	CGGAATTCCCAACCGGCTCGCCCACCACCTCAT<br>GATAAGCTTCCTCGAACAGGTGCGTCTGCAAGCT
Complete fragment of *pdaps*	GTGACGGGCGGGACCGTCGCTGACCG<br>TCACCGTGCAGGACTGGTGGGGTGGG
pdaps with its own promotor	ATGGATCCAGCGCCAGTTGTCCCAGTGGACCTC<br>GATAAGCTTTCACCGTGCAGGACTGGTGGGGTG
A domain of *pdaps*	CGGAATTCGTGACGGGCGGGACCGTCGCTGA<br>GATAAGCTTGGACGTCGAGGAACGGCGGCACCATG
njxA	CGGAATTCGATGACCATCATCTCCGTTCCCA<br>GATAAGCTTTCAGGCCGTCAGGTCCGTGGC
njxB	CGGAATTCGGTGACCACCACACGTCCCGCC<br>GATAAGCTTTCAGCCGTAGCGGCGTAGCTC

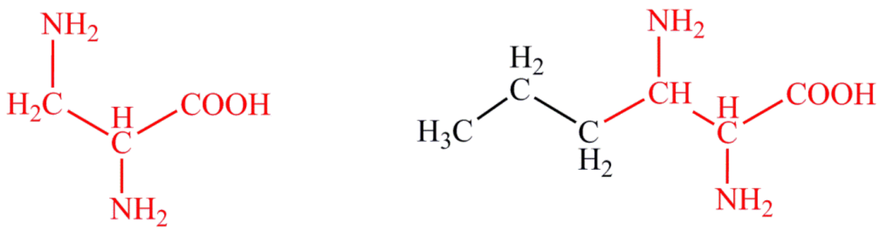

L-DAP **β-Lysine**

Supplementary Fig. S1. Structures of L-DAP and β-lysine

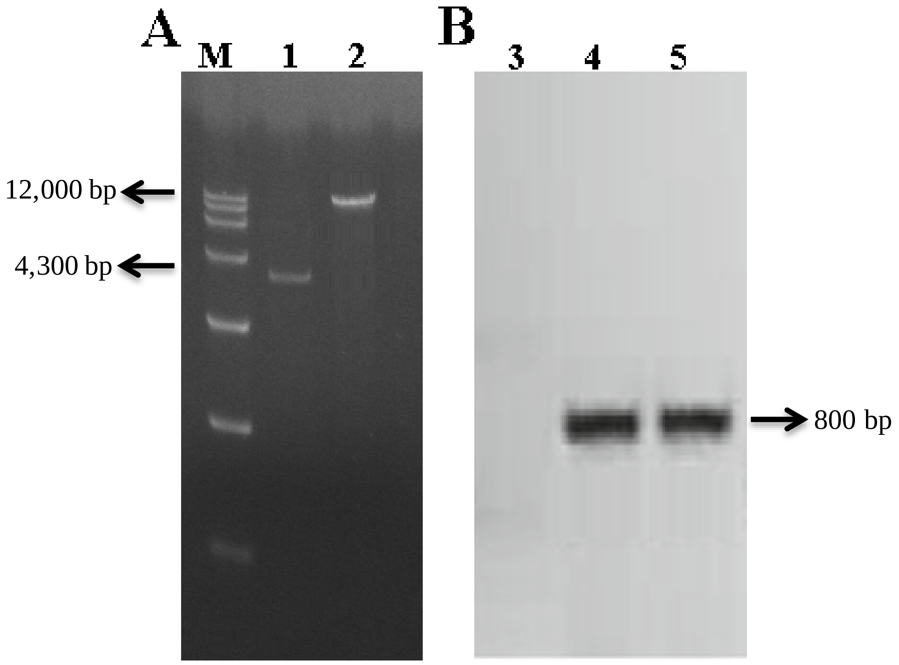

Supplementary Fig. S2. Analysis for putative *pdaps* disruption by PCR (A) and Southern-blot (B). For PCR analysis, the start and the end sequences of putative *pdaps* were used as primers for confirmation of the *pdaps* disruption. 1. using the chromosome of wild-type strain *S. albulus* PD-1 as the PCR template; 2. using the chromosome of putative *pdaps* disruption strain *S. albulus* PD-3 as a template. For Southern-blot analysis, the genomes were digested by *Sac*I and apramycin resistance gene was used as a probe. 4. wild-type strain *S. albulus* PD-1; 5 and 6. putative *pdaps* disruption strain *S. albulus* PD-3.

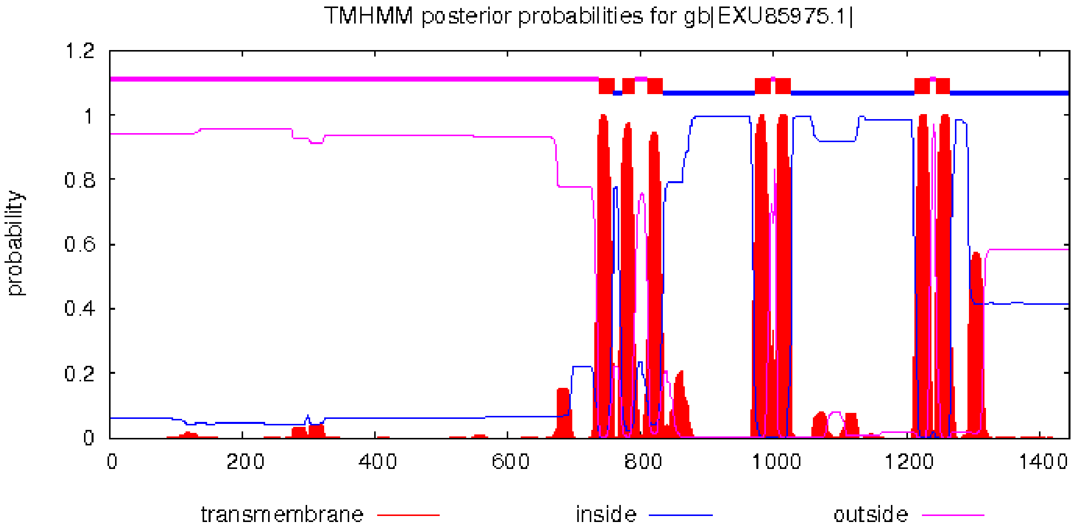

Supplementary Fig. S3. Analysis of transmembrane helices in PDAPs

A

```
PDAPs_C1.seq     ....................................................YLRLLGAR
PDAPs_C2.seq     CAKRLVLPRTPVGTHPVRSALGVRKWLSDKLLESSLTFT..NSLYATLYTVPWLRLLGAR
PDAPs_C3.seq     .LKRLVIGRYRPRTEPLWSWFVRRSEFVTGLYEAAAVPVGLAPLTGTPFLPPALRLFGAR
Consensus                                                             lrl gar

PDAPs_C1.seq     IGPRTTIATGVVPLPALLRIGADAAIGYGVTLRAWRVADGWVTVAPIDIGPRAYVGAHAV
PDAPs_C2.seq     IGPGAEVSTAAHLDPDLLTLGEGSFVADMAGVGGATFAGGRVTCRRTEVGRRAFLGNAAY
PDAPs_C3.seq     IGRRTWLGTTFLTEFDLVDIGDDAAIGAGVSLQTHLFEDRVMKMSKVTVQAGASVGSRSV
Consensus        ig      t       l   g                               a  g

PDAPs_C1.seq     LEPGARLAAGAGLGEQSVVGQGEVVPPGARWAGSPPAPVPTLDP..HVEDMLRAGGTTEH
PDAPs_C2.seq     LPAGTRTGPGSLIG.VGTVPPGDDVPPGTSWLGSPAIRLPQRQSSGTFPEELTFSPTRKA
PDAPs_C3.seq     VLYDGVVGEGVWLDALSLVMKGEYLTPGTAWRGIPVQGLALRVG.ARPSPELTPHPTSPA
Consensus                 g        v  g    pg  w g p                l    t

PDAPs_C1.seq     GRARHVAA
PDAPs_C2.seq     VRHRLAIE
PDAPs_C3.seq     R.......
Consensus
```

B

```
PDAPs_C1.seq     .....................................................YLRLLGA
Pls_C1.seq       ALAARALAGTIKPGAYPRGGATHLRLWTAERVVAAFGVP....SLLGTPWARLYARSLGC
PDAPs_C2.seq     .CAKRLVLPRTPVGTHPVRSALGVRKWLSDKLLESSLT..FTNSLYATLYTVPWLRLLGA
Pls_C2.seq       ...VRLLGRGITPGLHPASGGVAWRAWLVTRLLDGARG..SLFPLYASLGTPHWLRLLGA
PDAPs_C3.seq     ..LKRLVIGRYRPRTEPLWSWFVRRSEFVTGLYEAAAVPVGLAPLTGTPFLPPALRLFGA
Pls_C3.seq       .LAKWLLVGRFTVSEHPLWSSFVWRNELYDTFVESLAVPSMAGAFTGTPVLNWWLRTLGA
Consensus                                                               r  g

PDAPs_C1.seq     RIGPRTTIATGVVPLPALLRIGADAAIGYGVTLRAWRVADGWVTVAPIDIGPRAYVGAHA
Pls_C1.seq       ATGRNVALHT.MPPVTGLAELGDGCSVEPEADISGWWLDGDTLHIGAVRIGAGARVAHRS
PDAPs_C2.seq     RIGPGAEVSTAAHLDPDLLTLGEGSFVADMAGVGGATFAGGRVTCRRTEVGRRAFLGNAA
Pls_C2.seq       KVGRHAEISTVLPL.PSLLHVEDGAFLADDTLVAPFELRGGWLRLGTVRIGRRAFVGNSG
PDAPs_C3.seq     RIGRRTWLGTTFLTEFDLVDIGDDAAIGAGVSLQTHLFEDRVMKMSKVTVQAGASVGSRS
Pls_C3.seq       KIGRGVWLESYWLPETDLITVADGVSVNRGCVLQTHLFHDRIMRLDTVRLAEGSSLGPHG
Consensus          g              l

PDAPs_C1.seq     VLEPGARLAAGAGLGEQSVVGQGEVVPPGARWAGSPPAPVPTLDPHVEDMLRAGGTTEHG
Pls_C1.seq       MLMPGAVVGQGAELASGACLD..GEIPDGASWSGSPARPAG..AAERMAGAAWPAPAWQR
PDAPs_C2.seq     YLPAGTRTGPGSLIGVGTVPP.GDDVPPGTSWLGSPAIRLPQRQSSGTFPEELTFSPTRK
Pls_C2.seq       IVDPGHDVPDHSLVGVLSNAP.ADGEP.GSSWLGRPAMPLP.RVATQADPAR.TFAPPRR
PDAPs_C3.seq     VVLYDGVVGEGVWLDALSLVMKGEYLTPGTAWRGIPVQGLALRVGARPSPELTPHPTSPA
Pls_C3.seq       IVLPGTEVGARASIAPSSLVMRGESVPAHTRWAGNPIAGERP...ARPVPARAEGGAAA.
Consensus                                       w g p

PDAPs_C1.seq     RARHVAAA
Pls_C1.seq       SRRWSAAY
PDAPs_C2.seq     AVRHRLAI
Pls_C2.seq       LVRARAAV
PDAPs_C3.seq     R.......
Pls_C3.seq       ........
Consensus
```

Supplementary Fig. S4. (A) Alignment of the three tandem domains of PDAPs. (B) Alignment of the amino acid sequences between the tandem domains of PDAPs and Pls.

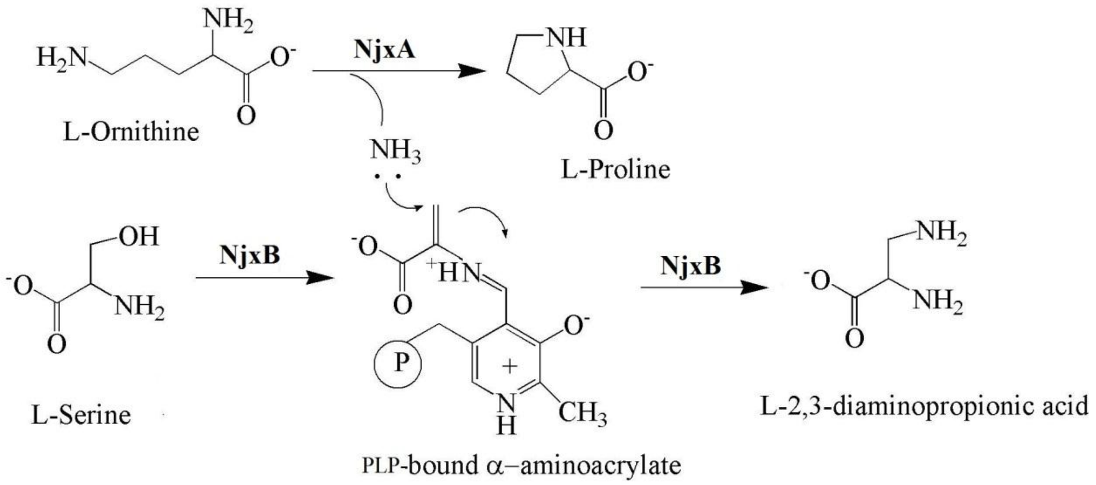

Supplementary Fig. S5. The putative synthesis mechanism of L-DAP in *S. albulus* PD-1.
